# *Lactobacillus* Re-Engineers Gut Microbiota to Overcome *E. coli* Colonization Resistance in Mice

**DOI:** 10.3390/vetsci12050484

**Published:** 2025-05-16

**Authors:** Jianlei Jia, Pengjia Bao, Qinran Yu, Ning Li, Hao Ren, Qian Chen, Ping Yan

**Affiliations:** 1 Key Laboratory of Yak Breeding Engineering Gansu Province, Lanzhou Institute of Husbandry and Pharmaceutical Sciences, Chinese Academy of Agricultural Sciences, Lanzhou 730050, China; jiajianlei87@163.com (J.J.); baopengjia@caas.cn (P.B.); yuqinran07@163.com (Q.Y.); 2 School of Life Sciences, Qilu Normal University, Jinan 250200, China; chenqian910@sohu.com; 3 Institute of Western Agriculture, The Chinese Academy of Agricultural Sciences, Changji 831100, China; lining06@caas.cn; 4 Teamgene (Shandong) Agricultural Technology Co., Ltd., Zibo 255086, China; renhao997@163.com

**Keywords:** *Lactobacillus*, *E. coli* infestation, colonization resistance, gut microbiota, barrier function

## Abstract

The intestinal health and functionality of animals play pivotal roles in nutrient digestion and absorption, as well as in maintaining defense against pathogenic invasions. These biological processes are modulated by various determinants, including husbandry conditions, dietary composition, and gut microbial ecology. The excessive use of anthropogenic antibiotics may disrupt intestinal microbiota composition, potentially leading to dysbiosis that directly compromises host homeostasis. While *Lactobacillus* species are recognized for their immunomodulatory properties, their precise mechanisms in regulating host anti-inflammatory gene expression and influencing mucosal layer maturation, particularly regarding *E. coli* colonization resistance, require further elucidation. To investigate the regulatory mechanisms of *Lactobacillus* in relation to intestinal architecture and function during *E. coli* infection, we established a colonic infection model using Bal b/c mice, conducting systematic analyses of intestinal morphology, inflammatory mediator profiles, and microbial community dynamics. Our results demonstrate that *Lactobacillus* supplementation (*Pediococcus acidilactici*) effectively mitigated *E. coli* O78-induced enteritis, with co-administration during infection facilitating the restoration of physiological parameters, including body mass, intestinal histoarchitecture, and microbial metabolic functions. Microbiome profiling revealed that the *Lactobacillus* intervention significantly elevated *Lactococcus* abundance while reducing *Weissella* populations (*p* < 0.05), concurrently enhancing metabolic pathways related to nutrient assimilation and environmental signal processing (including translation mechanisms, ribosomal biogenesis, amino acid transport metabolism, and energy transduction systems; *p* < 0.05). Mechanistically, *Lactobacillus* administration attenuated *E. coli*-induced intestinal pathology through multiple pathways: downregulating pro-inflammatory cytokine expression (IL-1β, IL-1α, and TNF-α), upregulating epithelial junctional complexes (Occludin, Claudin-1, and ZO-1), and stimulating mucin biosynthesis (MUC1 and MUC2; *p* < 0.05). These modifications collectively enhanced mucosal barrier integrity and promoted epithelial maturation. This investigation advances our comprehension of microbiota–host crosstalk during enteropathogenic infections under probiotic intervention, offering valuable insights for developing novel nutritional strategies and microbial management protocols in animal husbandry.

## Simple Summary

*Lactobacillus* is recognized as one of the most pivotal beneficial microbiota residing in the gastrointestinal ecosystems of animals, exhibiting multifaceted health-promoting effects. These biological advantages encompass regulating gut microbiota composition, stimulating intestinal motility, maintaining microecological homeostasis, mitigating inflammatory cascades, and improving gastrointestinal functional capacity. Our experimental evidence revealed that concurrent administration of *Lactobacillus* during *E. coli* colonization promoted the restoration of core physiological parameters, including body mass equilibrium, digestive enzymatic activity, intestinal histoarchitecture, and microbial metabolic functions. This therapeutic strategy consequently elevated endogenous probiotic populations while suppressing pathogen-induced inflammatory signaling pathways. Furthermore, microbiome analysis demonstrated that *Lactobacillus* supplementation significantly increased beneficial symbiont populations through enhancing nutritional metabolic networks (particularly amino acid transport and energy conversion pathways) and environmental signal transduction systems (*p* < 0.05), while simultaneously advancing mucosal layer maturation. Crucially, we established that *Lactobacillus* intervention ameliorated *E. coli*-induced enteric dysfunction and reinforced intestinal barrier integrity through microbiota-mediated modulation of epithelial tight junction complexes and mucin biosynthesis mechanisms (Occludin, Claudin-1, ZO-1, MUC1, and MUC2; *p* < 0.05).

## 1. Introduction

As the most important digestive and immune organ, the animal gut plays a vital role in protecting health and growth and development. It is an organ that is connected to the external environment organ and is exposed to various potentially harmful substances and pathogens which increase the risk of disease [1,2]. Pathogens such as viruses, bacteria, and parasites are the main infectious factors in animal intestinal diseases [3]. *Escherichia coli* (*E. coli*), a pathogenic bacterium, can enter hosts through fecal contamination originating from animal waste, subsequently leading to infection after traversing the intestinal system. This bacterium is capable of causing serious infectious diseases in both animals and humans [4,5]. As a microorganism extensively found in natural environments, *E. coli* can quickly disseminate among various hosts and settings [6]. The gut mucus layer plays a vital role in the gut’s chemical defenses, serving as a barrier against the adherence and penetration of pathogens [7]. The widespread transmission of highly pathogenic or antibiotic-resistant *E. coli* between humans and animals through food and environmental exposure poses a significant threat to human health, as well as to the economic sustainability of the livestock and poultry sectors. *E. coli* invasion can lead to a decrease in gut pH, disrupting the secretion and composition of gut mucus, ultimately diminishing its protective functions, hindering the proliferation of beneficial gut microbes, and compromising barrier integrity [8]. The complexities of livestock feeding practices and high population densities render animals particularly vulnerable to *E. coli* invasion, potentially resulting in severe health issues such as diarrhea and synovitis. Therefore, investigating techniques and mechanisms for preventing and treating *E. coli* invasions in animals holds considerable theoretical and practical significance for the field of animal health.

Antibiotics are extensively utilized as the primary strategy to combat *E. coli* outbreaks in contemporary livestock and poultry sectors, and their judicious application can effectively prevent various diseases [9]. However, the inappropriate or excessive use of antibiotics in animal husbandry exacerbates the growing issue of antibiotic resistance among animals, while also negatively impacting the pursuit of sustainable and high-quality farming practices [10]. The misuse of these medications, coupled with the persistence of antibiotic residues in agricultural products, has led to the emergence of antibiotic-resistant strains which may disrupt gut microbiota and trigger severe allergic reactions [11]. Recently, antimicrobial resistance has surfaced as a critical public health concern, with the over-utilization of antibiotics identified as a significant contributing factor. Resistance can develop through gene mutations or horizontal gene transfer [12]. The increasing prevalence of multi-drug-resistant *E. coli* strains, along with the continuous expansion of the resistance spectrum, presents substantial challenges for livestock production [13]. Consequently, efforts to mitigate *E. coli* resistance have become increasingly imperative. Research has demonstrated that *Lactobacillus* species play a crucial role in maintaining gut health and supporting the immune system in animals, owing to their antimicrobial and anti-inflammatory properties; for example, symbiotic gut microbiota, like those of humans and other mammals, contribute to functions such as growth and development, behavior, and physiology and play a key role in maintaining homeostasis [14].

*Lactobacillus* species are non-toxic, biodegradable, and biocompatible, rendering them suitable alternatives to conventional antibiotics. Recent studies have indicated that *Lactobacillus* species rank among the most significant beneficial bacteria present in the gastrointestinal tracts of animals, offering a diverse array of health benefits. These benefits include the regulation of gut microbiota, stimulation of intestinal peristalsis, preservation of the gut’s microecological balance, alleviation of inflammatory responses, and enhancement of overall gut functionality [15]. *Lactobacillus* contributes to the equilibrium of gut microbiota by promoting the proliferation of beneficial microorganisms while inhibiting the establishment of harmful ones [16]. Additionally, *Lactobacillus* plays a vital role in modulating the secretion of gut mucus; increasing the thickness of the mucus layer; producing metabolites such as organic acids and hydrogen peroxide; and restricting the growth of pathogenic bacteria, including *E. coli*, by competing for nutrients [17,18]. Moreover, *Lactobacillus* produces specific adhesion factors that enhance its ability to adhere to gut epithelial cells [19]. *Pediococcus acidilactici* belongs to one type of *Lactobacillus*, which can regulate the gastrointestinal flora and maintain the microecological balance of the intestinal tract. It has an antagonistic effect on pathogenic microorganisms in animals and can competitively inhibit pathogenic microorganisms, enhance the immune function of the animal body, produce beneficial metabolites, activate the activity of acid protease, participate in the body’s metabolism, and prevent the generation of harmful substances, which influences the gut immune response by activating immune signaling pathways in gut epithelial cells, thereby promoting the growth and differentiation of immune cells while also modulating their functions [20,21]. It aids in the repair and growth of gut epithelial cells, enhances the intestinal immune system, and improves gut barrier functionality by strengthening the tight junctions between epithelial cells, increasing mucus production, and balancing the gut microbial population.

This research utilized SPF-grade Balb/c mice to establish a model of *E. coli* infection, focusing on a systematic examination of gut morphology, expression of gut barrier function factors, and dynamics within the microbiota community. Our objective was to investigate the influence of *Lactobacillus* on the alterations in both the gut microbiome and gut barrier dysfunction induced by *E. coli* infection in mice. The results indicate that the molecular pathways through which *Lactobacillus* enhances gut physiological metabolism involve modulation of gut microbiota composition and regulation of host barrier function expressions. This insight may provide a foundational basis for the development of feeding strategies and management practices for animals.

## 2. Materials and Methods

### 2.1. Ethics Statement

All procedures involving the use of animals were approved by the Animal Care Committee at the Lanzhou Institute of Animal Science and Veterinary Pharmaceutics, Chinese Academy of Agricultural Sciences, China (202402131-03). Furthermore, approval for the slaughtering of the animals was obtained in accordance with the National Administration of Experimental Animal Slaughtering and Quarantine Regulations.

### 2.2. Animal Models and Experimental Design

Eighty Bal b/c mice, aged 6–8 weeks and weighing between 18 and 22 g, were obtained from the Laboratory Animal Center at Lanzhou Veterinary Research Institute in Lanzhou, China (vertebrate animal licensing, grant no. 2019-2025-07659). These mice were randomly assigned to standard plastic cages, with five animals per cage, separated by gender. The rodents were allowed to acclimate for two weeks in their new environment. All subjects received the D12450B Mouse Diet (Jiangsu). The facilities housing the mice maintained a consistent 12 h light–dark cycle, along with stable temperature and humidity levels of (23 ± 1) °C and (55 ± 5)%, respectively. The mice were kept in accordance with established animal care protocols, which included free access to water.

The experimental mice were divided into four distinct groups (n = 20 for each group, with an equal number of males and females) as detailed below: a control group (CG; each mouse received 6 g of the D12492 diet daily), a *Lactobacillus* group (LG; each mouse was administered 6 g of the D12492 diet daily, with the *Lactobacillus* treatment group receiving a 0.02% (*w*/*v*) *Lactobacillus* solution via intragastric administration throughout the experiment), a *Lactobacillus* with *E. coli* infestation group (AG; each mouse consumed 6 g of the D12492 diet daily. *E. coli* infections were instigated by administering 1 × 10^9^ CFU/mouse in 0.1 mL of LST (Lauryl Sulfate Tryptose) broth medium orally along with the diet. *Lactobacillus* administration in conjunction with *E. coli* infestation was provided to these mice by giving them a 0.02% (*w*/*v*) *Lactobacillus* solution intragastrically for the entire experimental duration), and a *Lactobacillus* treatment alongside *E. coli* infestation group (TG; each mouse received 6 g of the D12492 diet daily. *E. coli* infections were orally administered at 1 × 10^9^ CFU/mouse in 0.1 mL of LST broth medium along with the diet, followed 24 h later by *Lactobacillus* treatment through intragastric delivery of a 0.02% (*w*/*v*) *Lactobacillus* solution for the remainder of the experimental period). To assess the effect of the solvent, the control group was administered the same volume of sterile H_2_O intragastrically throughout the experiment. The *E. coli* O78 strain was sourced from diarrhea-afflicted mice and provided by the School of Life Sciences laboratory (Qilu Normal University), and the *Lactobacillus* (*Pediococcus acidilactici*) was purchased from Lallemand Inc. (Beijing, China) as a commercial strain (CNCM I-4622, BACTOCELL).

### 2.3. Sample Collection

To ensure cleanliness when managing the different experimental groups, 70% ethanol was used to disinfect gloves and surfaces, while clean paper towels were employed to collect freshly produced fecal pellets.

The *E. coli* O78 strains were reanimated, inoculated on LST Agar, and cultured at 37 °C for 24 h. Single colonies were then transferred to LST broth and cultured overnight in a shaker incubator at 37 °C. Strain detection employed Eosin–Methylene Blue Agar, and the colony-forming unit (CFU) counting method was used to determine the required strain concentration. *Lactobacillus* was reanimated and inoculated on MRS Agar medium, and a single colony was selected and inoculated into MRS liquid medium at 37 °C for 24 h. Strain concentration was determined using the CFU counting method.

All mice were subjected to weighing under a fasted condition after three weeks. The initial body weight (IBW) and final body weight (FBW) were accordingly registered. The weight gain rate (WGR) formula was “WGR (%) = (FBW − IBW)/IBW”.

Body weight measurements of the mice were recorded after the end of the experiment. The mice were sacrificed by cervical dislocation. The subjects were then positioned on a dissecting plate in a stable manner. The abdomen was promptly opened to carefully extract the jejunal tissues. Following rinsing with PBS, a portion of the jejunal tissues was preserved in 4% neutral buffered formalin for subsequent tissue section analysis, while another portion was immediately frozen in liquid nitrogen for later RT-PCR analysis. For each group, at least eight composite samples of jejunal mucosa tissue and digesta were collected for further sequencing analysis.

### 2.4. Sample Measurement

The level of *E. coli* present in the feces was determined by collecting freshly expelled fecal pellets into 1.5 mL centrifuge tubes containing phosphate-buffered saline (PBS). The samples were maintained on ice prior to thorough mixing using a Vortex Genie 2 (Scientific Industries) equipped with a horizontal microtube holder (Scientific Industries), in accordance with the protocol established by Rogers [22]. The mixing process was conducted at peak vortex speed for 5 min, or until the fecal pellets were fully homogenized. Strain concentration was determined using the CFU counting method with Eosin–Methylene Blue Agar.

The isolated jejunal tissues were preserved in a 4% buffered formaldehyde solution for 72 h. Following this, the specimens underwent dehydration using varying concentrations of glucose and were embedded in a frozen embedding medium and subsequently sliced into serial sections of 5–7 µm transversely. These sections were stained with hematoxylin and eosin (HE), which highlighted the villus height, crypt depth, and mucosal thickness.

Total DNA extraction from bacteria present in composite samples of jejunal mucosa tissue and digesta, as well as the construction of libraries and sequencing of the microbiome, was conducted by Shanghai Majorbio Bio-pharm Technology Co., Ltd. (Shanghai, China). To analyze microbial diversity and community composition, sequencing of the 16S rRNA gene was performed using the Illumina NovaSeq/Hiseq Xten (Illumina Inc., San Diego, CA, USA). The PCR amplification of the V3-V4 hypervariable region of the microbial 16S rRNA gene employed forward primers (5’-CCTACGGGNGGCWGCAG) and reverse primers (5’-GGACTACHVGGGTATCTAAT). The cycling conditions included an initial denaturation step at 95 °C for 2 min, followed by 35 cycles consisting of denaturation at 95 °C for 2 min, annealing at 72 °C for 30 s, and a final extension at 72 °C for 5 min.

A relative quantitative real-time PCR (qPCR) was performed to evaluate the copy numbers of the target genes. The primers were produced by Shanghai Biological Engineering Ltd. in China. The transcriptional levels of the target genes were normalized against the mRNA levels of glyceraldehyde-3-phosphate dehydrogenase (GAPDH). Primers for the target genes were designed using the Primer 5.0 Program and synthesized by Shanghai Biological Engineering Ltd. (Table 1).

### 2.5. Statistical Analysis

The analysis of 16S rRNA was conducted using R software (version 3.1.2) in conjunction with QIIME software (version 1.9.1) and UPARSE software (Uparse v7.0.1001). Following the UPARSE pipeline, multiplexed reads were combined into operational taxonomic units (OTUs) based on 97% sequence similarity. The classification of the 16S rRNA gene sequences was performed using the RDP Classifier (version 2.2). The OTUs were assessed using various metrics for alpha diversity analysis, which included OTU rank curves, rarefaction, and indices such as Shannon, Chao1, Simpson, and ACE. For beta diversity analysis, principal coordinate analysis (PCoA) and the unweighted pair-group method with arithmetic mean (UPGMA) were conducted using the weighted UniFrac distance in QIIME. Finally, PICRUSt was utilized to predict microbial functionality. Bacterial domains, phyla, and genera were compared using the Wilcoxon rank-sum test, with a false discovery rate (FDR)-adjusted *p* value of less than 0.05 designated as significantly different.

Prior to conducting any statistical analyses, all data underwent a thorough examination for outliers. The data were either outlined or evaluated using box-and-whisker plots alongside the Shapiro–Wilk test to confirm their normal distribution. The results of the analysis were expressed as means ± SEMs. For analysis, a complete randomized experiment design was employed utilizing the Statistical Analysis System (SAS) software, version 19.0 (SAS Inc.; Cary, NC, USA). To identify significant differences among groups, the LSR-SSR test was applied for normally distributed data, otherwise the Kruskal–Wallis test was used. A significance level of *p* < 0.05 was established, with *p* < 0.1 indicating a trend.

## 3. Results

### 3.1. E. coli O78 Strain Overcomes Microbiota-Mediated Growth Inhibition

**Figure 1:**
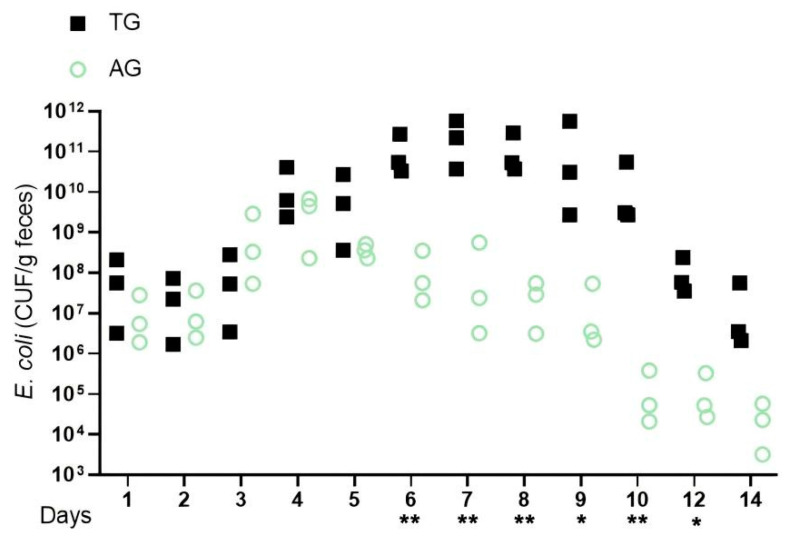
*E.coli* infection increases absolute pathogen abundance in the feces. Notes: *Lactobacillus*-free (Bal b/c) mice were infected with *E. coli* and subsequently treated with *Lactobacillus* (AG and TG). The graph shows colony-forming units (CFUs) of each strain recovered from feces at the indicated days after infection (days p.i.). * indicates a significant difference (*p* < 0.05) between A and B, ** indicates an extremely significant difference (*p* < 0.01) between A and B, and a blank indicates no significant difference between A and B (*p* > 0.05). A was AG, B was TG.

To develop a model using *Lactobacillus*-free mice infected with *E. coli* and subsequently treated with *Lactobacillus*, Balb/c mice were regularly monitored for the absence of enterobacterales. Based on the experimental design requirements, four distinct treatment groups of mice were established. Fecal samples were collected daily over a period of two weeks to assess the *E. coli* populations. Although pathogen counts in the feces initially decreased, a significant increase was observed three days post-infection. Following this, elevated levels of *E. coli* were detected in the feces of the infected groups (AG and TG), with the quantity in TG being notably higher than that in AG at the same time point. The increase in fecal pathogens in AG was more gradual, exhibiting a slow downward trend five days post-infection, after which the number of pathogens decreased to a less significant level ten days post-infection. In contrast, the rise in fecal pathogens within TG was rapid, resulting in the death of five mice. Notably, with the introduction of *Lactobacillus*, a gradual decline in pathogen counts was observed eight days post-infection, eventually dropping to a less influential level twelve days post-infection (Figure 1). These findings indicate that pronounced intestinal inflammation becomes evident shortly after *E. coli* infection, and the addition of *Lactobacillus* may aid in restoring gut structure, thereby mitigating the detrimental effects on the barrier. Overall, these findings suggest that the gut microbiota initially assists in reducing the pathogen load in the feces during the first two days following infection; however, by day three, the pathogen exploits its virulence factors to overcome this inhibition. The presence of *Lactobacillus* and its metabolites appears to alleviate gut inflammation induced by these virulence factors, which disrupts the *E. coli* colonization resistance within the gut’s growth environment.

### 3.2. A Change in Mouse Weight Is Linked to Lactobacillus Broken E. coli Colonization Resistance

Prior research has demonstrated that *Lactobacillus* can produce substances, such as organic acids and bacteriocins, which inhibit biomass and reduce colonization resistance against harmful bacteria like *E. coli* in the intestinal environment. This, in turn, affects the weight of mice. Supporting this notion, the body weight data presented in Table 2 indicate that the final body weight, weight gain, and weight gain rate in the *Lactobacillus*-treated group infected with *E. coli* were significantly lower than those in the other groups (*p* < 0.05). These results suggest that *Lactobacillus* may counteract the growth inhibition caused by *E. coli* through an unidentified mechanism, thereby effectively promoting weight gain.

### 3.3. E. coli Infestation and Lactobacillus Intervention Are Linked to Compositional Gut Microbiota Changes

**Figure 2:**
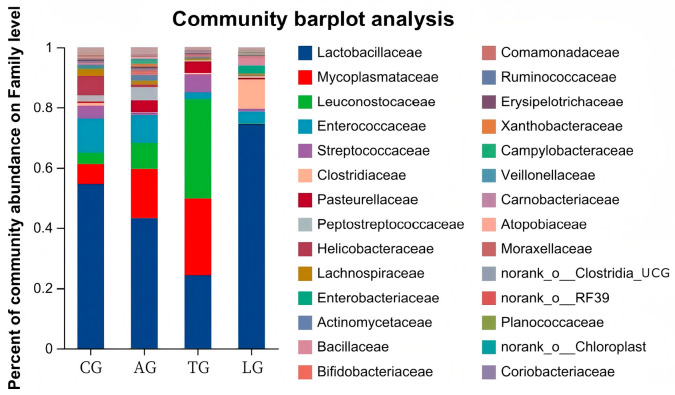
Community abundance percentages of microorganisms.

**Figure 3:**
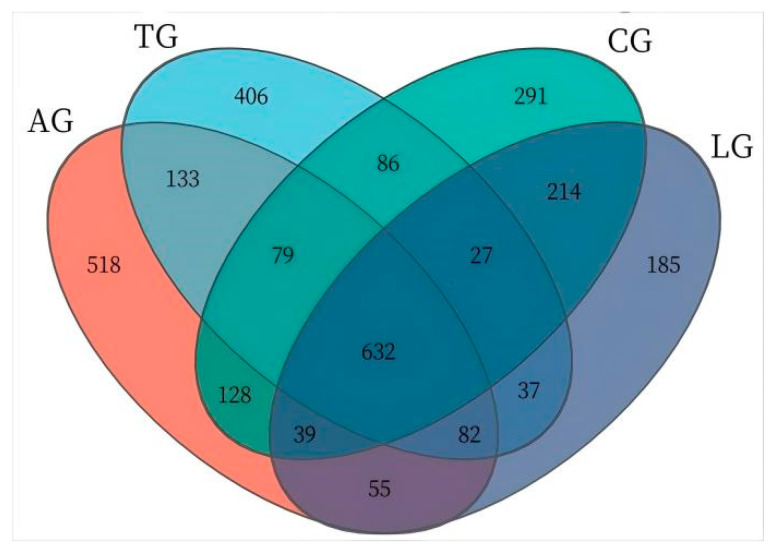
Jejunal sample microbiota Venn diagram. Note. The numbers was the OTU abundance, such as 632 was the shared OTUs of four group, 518 was the unique OTUs of AG.

The jejunum is an important organ for nutrition digestion, absorption, and metabolism and a barrier against harmful substances which plays an important role in animal health. Given that intestinal inflammation is known to alter the composition of the microbiota during *E. coli* invasion, to elucidate how *Lactobacillus* modulates microbial community dynamics during *E. coli*-induced intestinal inflammation, we conducted comprehensive 16S rRNA amplicon sequencing (V3–V4 region) on jejunal mucosa–digesta composites from four experimental cohorts. From 32 composite samples, we obtained 103,592 high-quality sequences with a Good’s coverage exceeding 99.2% across all groups, demonstrating sufficient sequencing depth. Taxonomic classification resolved 25 microbial families, with only 0.023 ± 0.003% of sequences remaining unclassified (Figure 2). Dominant taxa included *Lactobacillus* (39.2 ± 5.1%), *Weissella* (18.7 ± 3.8%), and the pathogenic genera *Mycoplasma* (7.3 ± 1.2%) and *Helicobacter* (4.9 ± 0.9%). Notably, *Lactobacillus* supplementation induced a significant taxonomic shift: the relative abundance of *Lactobacillus* increased by 32.4% (*p* < 0.05, Kruskal–Wallis test), while *Weissella* decreased by 14.2% compared to *E. coli*-challenged controls. This reciprocal relationship suggests competitive exclusion of potential pathogens through ecological niche occupation. Furthermore, the *Lactobacillus* + *E. coli* group (AG) exhibited 18.7% greater OTU richness than the *E. coli*-only groups (Figure 3), with the microbial community structure converging toward that of healthy controls (Bray–Curtis similarity index: AG vs. control = 0.78 ± 0.03 vs. 0.65 ± 0.05 in *E. coli* groups). These findings demonstrate that targeted *Lactobacillus* intervention preserves α-diversity and β-diversity metrics during pathogenic challenge, potentially through enhancing colonization resistance against Enterobacteriaceae expansion.

**Figure 4:**
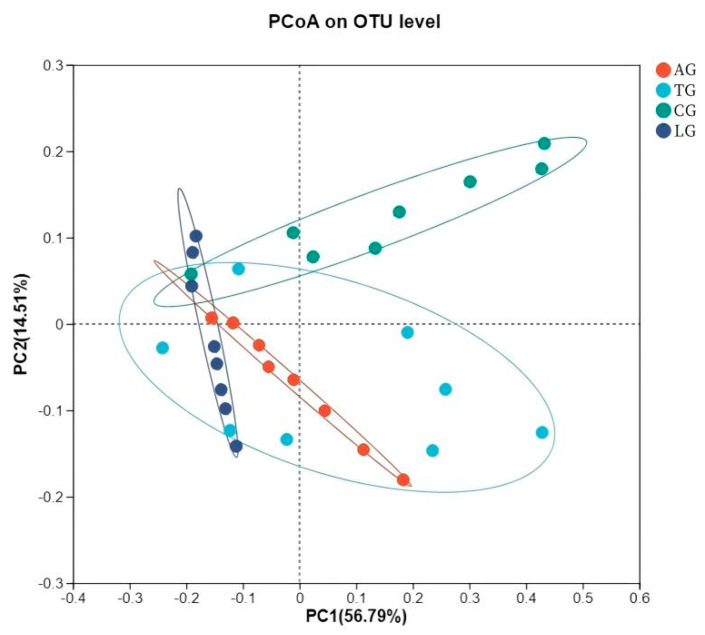
Jejunal sample microbiota bacterial PCoA analysis.

The jejunal sample microbiota assessment of alpha diversity was conducted using diversity indices (Shannon and Simpson) along with richness estimators (Chao1 and ACE). As shown in Table 3, the richness estimators (ACE and Chao1) increased for AG, TG, and LG compared to CG. However, the Simpson indices were significantly lower than that of CG (*p* < 0.05), while the Shannon indices were elevated in comparison to CG. Notably, AG and TG demonstrated greater vigor relative to LG. These findings suggest that the addition of *Lactobacillus* may enhance the richness and diversity of gut microbiota, particularly in the context of *Lactobacillus* intervention during *E. coli* infection. Furthermore, the beta diversity observed in LG was lower than that in TG and AG, suggesting a beneficial influence on gut microorganisms affected by *E. coli* infestation due to *Lactobacillus* supplementation. Principal coordinate analysis (PCoA) employing the weighted UniFrac similarity method revealed that PC1 and PC2 explained 56.79% and 14.51% of the total variance among the samples, respectively. This analysis illustrated that gut samples from distinct groups formed separate clusters in the ordination space (Figure 4), with microbiota composition displaying greater divergence following *E. coli* infection and *Lactobacillus* intervention compared to samples taken from CG. The microbiota composition exhibited increased divergence within the *Lactobacillus* treatment group alongside the *E. coli* infestation.

**Figure 5:**
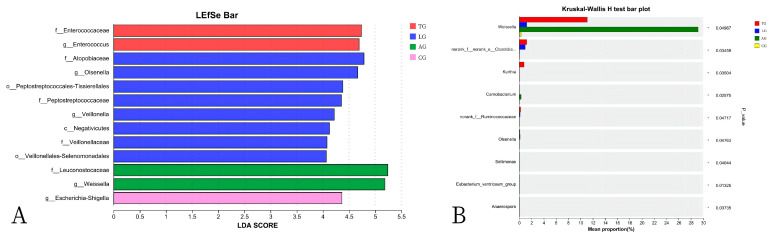
The linear discriminant analysis effect size (LEfSe) method identified the significantly different abundant taxa of bacteria ((**A**) shows the list of abundant bacterial taxa; (**B**) shows the significantly enriched bacteria).

**Figure 6:**
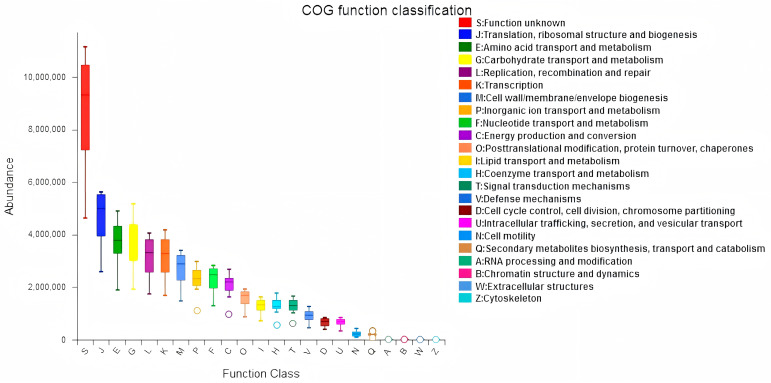
Jejunal sample microbiota COG analysis.

In this study, we examined the effects of *Lactobacillus* treatment on the alleviation of damage caused by *E. coli* infections with the objective of reducing the prevalence of spoilage microorganisms while fostering beneficial microbial populations in the gut environment three weeks post-treatment. The results from the LEfSe analyses, which aimed to emphasize the significance of various taxa (with a relative abundance exceeding 1%) across all experimental groups, as well as the LDA outcomes derived from the LEfSe analysis, are presented in Figure 5. In the control group (CG), Escherichia-Shigella was identified as the overwhelmingly dominant taxon (LDA = 4.36). In contrast, the *Lactobacillus* group (LG) exhibited notable predominance of taxa such as Atopobiaceae (LDA = 4.78), *Olsenella* (LDA = 4.67), Peptostreptococcales-Tissierellales (LDA = 4.38), Peptostreptococcaceae (LDA = 4.35), and *Veillonella* (LDA = 4.22). Within the antibiotic group (AG), Leuconostocaceae (LDA = 5.24) and *Weissella* (LDA = 5.18) were significantly prevalent. The treatment group (TG) showed a marked presence of Enterococcaceae (LDA = 4.74). The findings indicated significant enrichment of processes such as translation, ribosomal structure and biogenesis, amino acid transport and metabolism, and energy production and conversion (*p* < 0.05). Furthermore, notable differences were observed between AG and TG concerning inorganic ion transport and metabolism, coenzyme transport and metabolism, and other biological processes (*p* < 0.05, Figure 6). Additionally, the functional prediction analyses suggested that *Lactobacillus* treatment may positively affect gut microbiota function and enhance the gut barrier following *E. coli* infection.

### 3.4. Jejunal Inflammation Elicited During E. coli Infection

**Figure 7:**
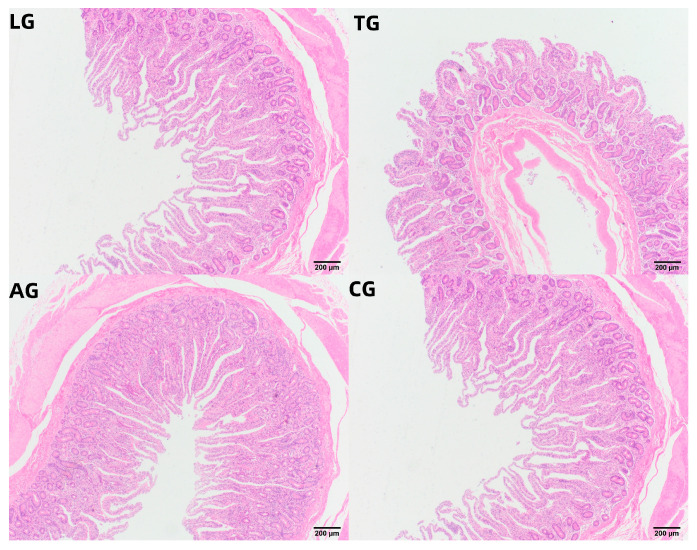
Jejunal samples of HE-stained tissue.

Given that intestinal inflammation is known to alter intestinal morphology during *E. coli* infection, we subsequently examined the morphological changes induced by *Lactobacillus* intervention over time. Histological analysis of jejunal tissue, focusing on villus height, crypt depth, and the villus height/crypt depth (V/C) ratio, was conducted three weeks post-*E. coli* infection across the various treatment groups, as summarized in Table 4 and Figure 7. The measurements of villus height and crypt depth in the LG group were significantly greater than those in the CG, AG, and TG groups (*p* < 0.05), while both CG and AG exhibited notably larger values than TG (*p* < 0.05). Mice in the AG group demonstrated some improvement in gut villus height and crypt depth, although these measures did not fully return to baseline levels. The V/C ratio in the TG, LG, and CG groups was lower than in the AG group (*p* < 0.05). Thus, we propose that *Lactobacillus* supplementation may facilitate the recovery of gut villus length and crypt depth adversely affected by *E. coli* infection, effectively improving both the villus height and crypt depth ratio, along with the maturation of the mucus layer.

**Figure 8:**
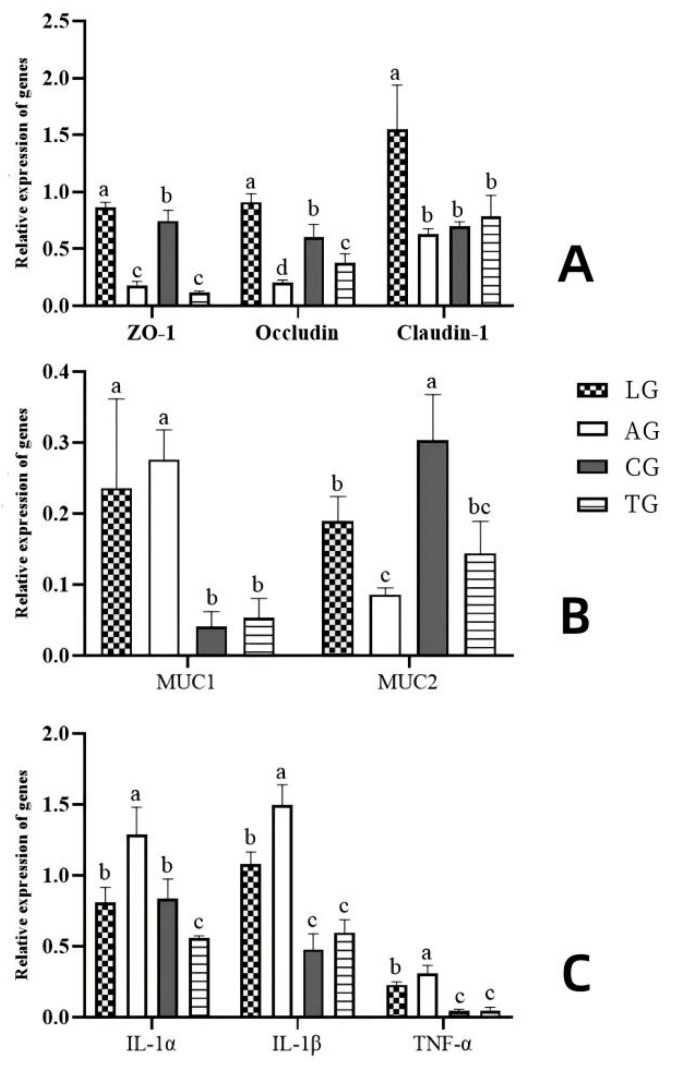
Effects of *Lactobacillus* supplementation on the target genes in *E. coli*-infected mouse guts ((**A**) shows the tight junction protein-coding genes, (**B**) shows the digestive metabolism protein-coding genes, and (**C**) shows the inflammatory factor protein-coding genes). Note. Different letters represent significant differences (*p* < 0.05), while the same letters represent no significant differences (*p* > 0.05).

To investigate gut barrier function in the context of *Lactobacillus* intervention during *E. coli* infection, we examined gene expression associated with relevant tight junctions, inflammatory markers, and digestive processes following various treatments. The gene expression analysis, conducted using reverse transcription polymerase chain reaction (RT-PCR, Figure 8), revealed that the transcription levels of inflammatory factor-related genes (IL-1β, IL-1α, and TNF-α) in AG were significantly lower compared to those in TG (*p* < 0.05). Concurrently, the expression of genes involved in tight junctions (Occludin, Claudin-1, and ZO-1) and digestive metabolism (MUC1 and MUC2) exhibited marked increases (*p* < 0.05). Our findings suggest that the transcriptional activity of genes linked to tight junctions, inflammatory responses, and digestive metabolism underwent significant changes during *Lactobacillus* intervention amidst *E. coli* infection, indicating that the addition of *Lactobacillus* could mitigate the damage inflicted by *E. coli* on gut barrier function.

## 4. Discussion

The health and functionality of an animal’s gut play a critical role in husbandry production. The gut microbiota is essential for preventing harmful elements from infiltrating the body, thereby influencing growth performance and disease resistance [23]. This microbiota comprises various microorganisms and their genes, with significant compositional changes often referred to as dysbiosis, which is characterized by the presence of potentially pathogenic microorganisms or a decrease in microbial diversity [24], anthropogenic activities [25], and pollutants [26]. This perspective directs research toward identifying correlations between changes in composition and function within the gut microbiota. Notable alterations in the host environment were observed three days post-*E. coli* infection, including increased fecal *E. coli* excretion and damage to the mucosal layer. After ten days of *E. coli* infection and *Lactobacillus* intervention, alterations in the host environment were recorded, marked by reduced pathogen proliferation, which suggests weakened colonization resistance, despite the absence of significant changes in the quantities of *E. coli* within the microbiota composition. Consequently, *Lactobacillus* may diminish resistance to *E. coli* colonization by inducing modifications in the host environment through metabolic reprogramming of the epithelial cells prior to evident shifts in the microbiota composition.

The integrity, both structural and functional, of the intestinal tract plays a crucial role in nutrient uptake, immune system development, and resistance to diseases [27]. Any modifications or dysfunctions within the intestinal architecture can negatively impact feed efficiency, overall productivity, and animal health. Acting as a “secondary genome” that influences the health characteristics of the host superorganism, the intestinal microbiota is closely linked to host nutrition, metabolic processes, and immune responses [28]. Pathogenic microorganisms can disrupt the functions of intestinal microbiota, permitting harmful substances to enter the animal’s body; this phenomenon is known as the breakdown of colonization resistance [29]. *Lactobacillus* is frequently regarded as a natural alternative to antibiotics and has been shown to inhibit the growth and reproduction of harmful microorganisms while preserving intestinal structure and promoting immune development [30]. In our study, we meticulously assessed anti-inflammatory properties, antioxidant capabilities, and digestive metabolic efficiency, leading to insights regarding the relationship between *Lactobacillus* supplementation and an *E. coli* infection model. The presence of *Lactobacillus* notably downregulated inflammatory markers while enhancing antioxidant levels. Furthermore, the addition of *Lactobacillus* resulted in a significant increase in gut digestive enzyme activity, indicating that it may provide protective benefits for the intestinal barrier and support the maturation of mucosal layer function. *Lactobacillus* demonstrates substantial potential as an effective agent for inhibiting and mitigating *E. coli*-induced damage, consistent with findings from earlier studies.

The gut microbiota plays a crucial role in the breakdown of resistant food components, such as dietary fiber, and is capable of modulating the metabolism of bile acids, lipids, and amino acids in the host [31]. It accomplishes this through the exchange of metabolites, engagement in signaling pathways, and influence on host gene expression and energy balance. Previous research has shown that *Lactobacillus* can promote the growth and repair of intestinal epithelial cells, regulate the secretion of intestinal mucus, increase the thickness of the mucus barrier, and enhance the functionality of the intestinal barrier [32]. Additionally, by competing for nutrients, including carbohydrates and amino acids, and producing specific metabolites, such as organic acids and hydrogen peroxide, *Lactobacillus* can inhibit the proliferation of *E. coli*, which may subsequently enhance animal growth and performance [33]. Our results indicated that the mice in LG experienced greater weight gain, showing a higher average daily increase compared to the other groups. In contrast, the body weight of mice in TG lagged behind that of the AG. This suggests that the inclusion of *Lactobacillus* in their diet could enhance the growth performance of mice to some extent. Furthermore, the gut microbiome enriched with *Lactobacillus* may regulate energy homeostasis in the guts of mice and maintain stability by performing essential functions and combating pathogens, for instance, optimizing nutrient extraction from the diet to maximize potential economic benefits.

Recent research has indicated that *Lactobacillus* plays a crucial role in preserving intestinal microbiota, which is essential for optimal gut health [34]. It enhances the diversity and abundance of beneficial bacteria that protect the gut barrier while inhibiting the proliferation of endotoxin-producing organisms [35,36]. Consequently, we propose that dietary modulation of gut microbial phylotypes through *Lactobacillus* supplementation may aid in alleviating dysbiosis induced by *E. coli* infection in host gut microbiota. Furthermore, the timing of *Lactobacillus* intervention significantly influences gut microbiota composition. At the phylum level, the study identified *Lactobacillus*, *Weissella*, *Mycoplasma*, *Enterococcus*, *Ureaplasma*, *Clostridium*, *Gallibacterium*, *Helicobacter*, and *Lactococcus* as the predominant phyla, collectively accounting for over 90% of the gut microbiota. These results suggest that the composition and functionality of the beneficial gut microbiota in the mice that received the *Lactobacillus* supplementation were superior to those of the mice on a standard diet. Our findings demonstrate that *Lactobacillus* can partially ameliorate gut microbiota disturbances caused by *E. coli* infection, with the microbiota composition of the *Lactobacillus*-supplemented mice aligning more closely with that of mice on a normal diet. Additionally, there was a notable decline in the relative abundance of *Weissella* in the gut microbiota of mice in the treatment group following *Lactobacillus* intervention, while the relative abundance of *Lactobacillus* significantly increased. A potential explanation for these findings may lie in the positive correlation between *Lactobacillus* and the metabolism of lipid-soluble nutrients. In contrast, dietary nutrients were not fully metabolized due to *E. coli* infestation, which led to the development of *Weissella*. This suggests that *Lactobacillus* intervention enhances the metabolism of dietary nutrients. The dominant microbial community identified between AG and TG included Escherichia-Shigella, Atopobiaceae, *Olsenella*, Peptostreptococcales-Tissierellales, Peptostreptococcaceae, *Veillonella*, Leuconostocaceae, *Weissella*, and Enterococcaceae. Our study demonstrated that *Lactobacillus* intervention significantly improved the diversity of gut microbiota. Notably, although the species within the dominant family remained unchanged, the ratios of dominant microbiota exhibited shifts. Consistent with our previous studies, published research has indicated that *Lactobacillus* intervention counteracts microbial community disorder resulting from *E. coli* infestation. Furthermore, functional predictions revealed substantial enhancements in nutrient metabolism pathways and environmental information processing due to *Lactobacillus* intervention. Our results indicate that *Lactobacillus* interventions contributed to a balanced gut microbiota in mice while also exhibiting antibacterial, anti-inflammatory, and digestibility–regulatory effects.

## 5. Conclusions

Our research indicates that the presence of *Lactobacillus* could alleviate gut inflammation induced by *E. coli* virulence factors and that concomitant feeding of *Lactobacillus* during the *E. coli* infestation process could facilitate the normalization of body weight, gut morphology, and microbial physiological function. This intervention leads to an increase in gut probiotics and a reduction in the host’s inflammatory response associated with *E. coli* infection. Furthermore, we confirmed that the introduction of *Lactobacillus* enhances the presence of beneficial microbial populations, which subsequently improves nutritional metabolism pathways and environmental signaling while promoting the development of the mucosal layer. Ultimately, we demonstrated that *Lactobacillus* intervention can partially alleviate gut dysfunction caused by *E. coli* infection and effectively strengthen the integrity of the barrier function in mice by modulating gut microbiota composition. This research enhances our understanding of host–microbe interactions in *E. coli*-infected animals treated with *Lactobacillus* and provides a theoretical foundation for the development of feeding strategies and animal management.

## Figures and Tables

**Table 1:** The primer sequences of target genes.

Gene Name		Primer Sequences (5′→3′)
ZO-1	Primer F	GTGGAATGATGTCGGAATA
	Primer R	CTACAATGCGGCGATAAA
Claudin-1	Primer F	TCTTCGACTCCTTGCTGAATCTGAAC
	Primer R	CCATCCACATCTTCTGCACCTCATC
Occludin	Primer F	TGGATCTATGTACGGCTCAC
	Primer R	CCATCTTTCTTCGGGTTT
MUC1	Primer F	AGCCACCAGTCCAGACCACAG
	Primer R	TAGGTAGCACCGAGGAGCCATTG
MUC2	Primer F	GCTGACGAGTGGTTGGTGAATG
	Primer R	GATGAGGTGGCAGACGGAGAC
IL-1β	Primer F	CCGTGGACCTTCCAGGATGA
	Primer R	GGGAACGTCACACACCAGCA
IL-1α	Primer F	CAAACTGATGAAGCTCGTCA
	Primer R	TCTCCTTGAGCGCTCACGAA
TNF-α	Primer F	CCCTCACACTCAGATCATCTTCT
	Primer R	GCTACGACGTGGGCTACAG
GAPDH	Primer F	AGGTCGGTTGTGACGGATTTG
	Primer R	TGTAGACCATGTAGTTGAGGTCA

**Table 2:** The body weight changes with *Lactobacillus* supplementation.

	CG	LG	AG	TG
Initial Body Weight (IBW, g)	19.20 ± 1.05	19.05 ± 0.75	18.95 ± 0.95	19.15 ± 1.20
Final Body Weight (FBW, g)	24.00 ± 0.95 b	26.30 ± 0.80 a	23.45 ± 1.00 b	22.45 ± 0.85 b
Weight Gain (WG, g)	4.80 ± 0.35 b	7.25 ± 1.15 a	4.50 ± 0.60 b	3.30 ± 0.95 c
Weight Gain rate (WGR, %)	24.87 ± 1.21 b	37.49 ± 0.97 a	24.15 ± 1.03 b	17.62 ± 1.07 c

Note. Different letters represent significant differences (*p* < 0.05), while the same letters represent no significant differences (*p* > 0.05).

**Table 3:** The jejunal sample microbiota alpha diversity of different treatment groups.

Treatment	ACE	Chao1	Shannon	Simpson
TG	278.92 ± 36.28 a	263.75 ± 13.52 a	0.32 ± 0.03 a	1.56 ± 0.27 b
LG	221.3 ± 29.61 b	225.17 ± 17.02 b	0.26 ± 0.05 a	1.18 ± 0.16 b
AG	287.06 ± 33.05 a	280.89 ± 17.09 a	0.29 ± 0.08 a	1.42 ± 0.28 b
CG	188.74 ± 21.05 c	179.29 ± 16.32 c	0.18 ± 0.04 b	2.82 ± 0.21 a

Note. Different letters represent significant differences (*p* < 0.05), while the same letters represent no significant differences (*p* > 0.05).

**Table 4:** Histological analysis of gut tissue morphology.

Treatment	Villus Height (VH)	Crypt Depth (CD)	VH/CD
TG	321.05 ± 19.05 c	165.85 ± 9.55 c	1.94 ± 0.25 b
LG	463.15 ± 13.35 a	220.50 ± 11.35 a	2.10 ± 0.12 b
AG	398.25 ± 11.55 b	165.75 ± 10.50 c	2.40 ± 0.23 a
CG	409.20 ± 15.60 b	198.05 ± 12.85 b	2.07 ± 0.43 b

Note. Different letters represent significant differences (*p* < 0.05), while the same letters represent no significant differences (*p* > 0.05).

## Data Availability

Merged DNA sequences were deposited in the Genome Sequence Archive with accession number PRJNA1203734 (http://www.ncbi.nlm.nih.gov/bioproject/1203734; 27 December 2024).
